# Supplementary material for: Patterns of Intron Gain and Loss in Fungi
Source: PLoS Biol. 2004 Nov 30;2(12):e422. doi: 10.1371/journal.pbio.0020422 (PMC532390; doi:10.1371/journal.pbio.0020422)
Supplement: Table S1 — Also available at http://genes.mit.edu/NielsenEtAl/. (4.3 MB ZIP). [file pbio.0020422.st001.zip › NielsenEtAl/html/253.html]

AN4761.1.NCU01912.1.MG02954.1.FG08935.1


```
 CLUSTAL W (1.82) Multiple Sequence Alignments - Introns Inserted


Sequence 1: NCU01912.1	1167 aa
Sequence 2: FG08935.1	960 aa
Sequence 3: MG02954.1	997 aa
Sequence 4: AN4761.1	918 aa
Alignment Length: 1206 aa
Number Identitical Residues: 486 aa
Alignment Score (without introns) 23924


MG02954.1 	MARQAVAKAAPAPMRAETPPIDKG-------GNKVAQKPKNKPKSKS-STDYTSEGVEDN
NCU01912.1	MARSGKKSPAPPPAYAPAYAPPPAYEAVP-TSAVVSKNKNNKNKSAKVKDDYASEGVQDN
FG08935.1 	MARTRGKSPQPPVKATTPVTEKQQPIAVPQQQQPTAVTTKSKNKNKNKVTSYQSEGVEDN
AN4761.1  	MAKDTLDVRPPRTSSRSPSR---------------SPRPKNRKKATAVASSYQSDGVTDN
          	**:       *      .                 :   :.: *      .* *:** **

MG02954.1 	DVFLLPISDYQWMFAITALAAAVRLFRIYQPTSVVFDEVH2FGGFASKYIKGRFFMDVHP
NCU01912.1	DPFLLPGSDFVLLTIITMLSAVVRLFRIYQPDSVVFDEVH2FGGFASKYIKGKFFMDVHP
FG08935.1 	DVFLLPLSDYWVVLALMALATVVRVYKIYQPTSVVFDEVH2FGGFATKYIKGKFFMDVHP
AN4761.1  	NIFDLPMSDYKVMVLVTIVAAVVRLFRIYQPTSVVFDEVH2FGGFATKYIKGRFFMDVHP
          	: * ** **:  :  :  :::.**:::**** ******** *****:*****:*******

MG02954.1 	PLAKLLITLAGWLAGFDGNFDFKEIGKDYLEPKVPYVAMRMFPAICGILLAPTMFLTLKA
NCU01912.1	PLAKLMITLFGWLAGFDGSFDFKEIGKDYVEPGVPYVAMRMFPAICGILLAPTMFLTLKA
FG08935.1 	PLAKMLIALTGWLAGFDGSFDFKEIGKDYIEPGVPYVAMRMFPAICGILLIPCMFFTLKA
AN4761.1  	PLAKLLITLAGWLAGFKGDFDFKEIGKDYLEPGVPYVAMRMLPAILGVLTVPIMFLTLKA
          	****::*:* ******.*.**********:** ********:*** *:*  * **:****

MG02954.1 	LGCRTIIASMGAGLIIFE1NGLLTQARLILLDSPLMITTAFSVLAFVSFTNQHEQGPSKA
NCU01912.1	AGCRSTTAFMGACLIIFE1NGLLTQARLILLDSPLMIGTALTALGFTAFTNQQELGPGKA
FG08935.1 	VGCRTMTATMGAGLIIFE1NGLLTQARLILLDSPLVAATAFTVLAFSCFTNQHELGPSKA
AN4761.1  	TGCRTNTAVLGAGAILFE1NGLVTQSRFILLDSPLVFFTALTALSFTCFTNQQELGPSHA
          	 ***:  * :**  *:** ***:**:*:*******:  **::.*.* .****:* **.:*

MG02954.1 	FGPSWWFWLAMTGLGLGANFSVKWVGLFTIAWVGALTLVQLWVLLGDTKNVTM0KTFYKH
NCU01912.1	FTPSWWFWLVVTGLGLGITASIKWVGLFTIAWVGSLTLIQLWVVLGDHKHVSP~RAFAKH
FG08935.1 	FQLSWWFWLVLTGLGLGITVSIKWVGLFTIAWVGSLTLVQLWVLLGDNKNVSM0RLFSKH
AN4761.1  	FRGPWWFWLVATGFSLGATLSVKWVGLFTVAWVGSLTILQLWVVLGDTNNVTP0RLWFKH
          	*  .*****. **:.** . *:*******:****:**::****:*** ::*:  : : **

MG02954.1 	FLARAVCLIMIPAGVYMGMFAIHFLCLVNPGEGDGFMSSEFQSTLNSKGMQDVPADVLMG
NCU01912.1	FMARAFCLIVIPLTFYMAMFGIHFMCLTNPGDGDGFMSSEFQSTLNSKGMQDVPADVLMG
FG08935.1 	FMARVFCLIVIPLTFYMAMFAIHFVCLQNPGDGDGFMSSEFQATLNNKRMKDVPADVMMG
AN4761.1  	FFARVFCLIVIPLAFYCSMFAIHFRCLVNPGEGDGFMSSEFQATLNSKGMAAVPADVVFG
          	*:**..***:**  .* .**.*** ** ***:**********:***.* *  *****::*

MG02954.1 	SRVAIRHLNTQGGYLHSHPLMYPTGSKQQQITLYPHKDENNLWFLENQTQPVDANGVQIN
NCU01912.1	SRVTIRHVNTQGGYLHSHPLMYPTGSKQQQITLYPHKDDNNIWLLENQTQPLGIDGQPIN
FG08935.1 	SRVSIRHVNTQGGYLHSHPLMYPTGSKQQQITLYPHKDDNNIWLLENQTQPLGIDGQPIN
AN4761.1  	SRLSIRHHNTQGGYLHSHSHMYPTGSKQQQITLYPHKDDNNLFIAENQTQPLDANGAEIP
          	**::*** **********. ******************:**::: ******:. :*  * 

MG02954.1 	GTNAWDKVTPLPYIQDGAVIRIYHIATHRRLHSHDVRPPVTEAEWQNEVSAYGYEGFDGD
NCU01912.1	GTGAWDHLPETPYIKDGAILRLYHLPTHRRLHSHDVRPPVTEADWQNEVSAYGYEGFDGD
FG08935.1 	GTKAWDALPEPVHIKDGAVLRLYHSPTHRRLHSHDVRAPITEADWQNEVSAYGYEGFEGD
AN4761.1  	GPFAWDNLTTN-YIEDGAVIRLHHLMTHRRVHSHNERPPVTDVDWQFEVSAYGYEGFPGD
          	*. *** :.   :*:***::*::*  ****:***: *.*:*:.:** ********** **

MG02954.1 	ANDFFRVEIVKKKSAKGAAQDRLRTLETKFRLVHVMTGCVLFSHKVKLPEWASEQQEVTC
NCU01912.1	ANDFFRVEIVKKKSKSGVAQERLRTIDTKFKLVHIMTGCVLFSHKVKLPTWASEQQEVTC
FG08935.1 	ANDLFRVEIVKKKTRGAVAKERLRTIESKFRLVHVMTGCVLFSHKVKLPDWASEQQEVTC
AN4761.1  	ANDYWRVEIVKSLSDGEEAKKRLRTIQSKFRLVHVMTGCVLFSHKVKLPDWGFDQQEVTC
          	*** :******. :    *:.****:::**:***:************** *. :******

MG02954.1 	ARGGTLPNSLWYIESNEHAQLNETAERVNYRNPGFLGKFIELNTVMWKTNAGLVESHAWD
NCU01912.1	AKGGSLPNSLWYIEFNDHPQLGADAEKVNYRNPGFFGKFWELQKVMWKTNAGLVESHAWD
FG08935.1 	ARGGTLPNSLWYIEGNTHPQLQGDVEKVNYRNPGFFGKFWELQKVMWRTNAGLTDSHAWD
AN4761.1  	AKGASLPNSIWYIESNKHPMLPPDAEKVTYRNPGFFGKFFELQKVMWTTNAGLTDSHAWD
          	*:*.:****:**** * *. *   .*:*.******:*** **:.*** *****.:*****

MG02954.1 	SRPDSWPLLKRGINFWGKERRQIYLIGNPVVWWSSTLAVVIYAVFKGIAVLRWQRNYKDY
NCU01912.1	SRPEAWPILRRGINFWGRQHRQVYLMGNPIIWWSATAAVVIYVLFKGIAILRWQRSCNDY
FG08935.1 	SRPESWPILRRGINFWGRQHTQVYLLGNPIIWWSSSVAVAIWIVFKAIAVLRWQRSCNDY
AN4761.1  	SRPPSWPTLLRGINFWGRDHRQVYLFGNPFVWYSSTLAVLVYVIFKGISLLRWQRNCGDY
          	*** :** * *******::: *:**:***.:*:*:: ** :: :**.*::*****.  **

MG02954.1 	NNVVFKRFDYEIGSSVLGWALHYFPFYLMQRQLFLHHYFPALYFAIMAWCQVMDFSLSRF
NCU01912.1	ANPIFKRFDYEVGTSVLGWALHYFPFYLMQRQLFLHHYFPALYFAIIAFCQVFDFVTARI
FG08935.1 	ANTTFKRFDYEIGTSVLGWALHYFPFYLMKRQLFLHHYFPALYFAVIALCQLFDYATARV
AN4761.1  	RNPAFKRFDYEIGTSVLGWGFHYFPFYLMARQLFLHHYFPALYFAILALAQEFDFITNRI
          	 *  *******:*:*****.:******** ***************::* .* :*:   *.

MG02954.1 	RGFGIRENPIIVRAAAVGFLA~ISIVAFLLLSPLAYGNTWTRPECQRVKLLSGWDFDCNA
NCU01912.1	S--LVKDKVIFNRLGAVLFLT~LSMVVFWLFSPLAYGNPWTKAECNRVKLFSTWDFDCNT
FG08935.1 	PGVGARETSVINRIATVSFLV~LSAAVFTLYSPLAYGSPWTKTDCKRVKLFTTWDFDCNT
AN4761.1  	R-----------------SLG1LTIFAFTVYSPLIYGNPWTRDACKQVKLLKSWDFDCNT
          	                   *  ::  .* : *** **..**:  *::***:. ******:

MG02954.1 	FPDT0YEAYQITTPQNPVVAQTPQQSVNAQPPPPQMKPKEDGGEKKDINQDQQQAVISGA
NCU01912.1	FLDS0YDNYYGITPTSQTPSQTPVQGTLERPA-----------VANAPPQAQKPIVNQEQ
FG08935.1 	FYDS0YEKYSEIPSISSSVVPTTSVAKKEEPK-----------KEVPVVQQQEEAVIS--
AN4761.1  	FYTD0LNQYVTHFSSVNSAVPTTAASIPQSPAP----------VKAEPKQEQKEQKEAKV
          	*     : *    .       *.  .    * .                * *:       

MG02954.1 	PNPALAVDNSEVLPGGQRVLSREERVEYRDDKGNLLNEEQVKAL--QGKVEFKTRYETRT
NCU01912.1	QAAKQEPSAN---PVGQRVISRQEKIEYRDQDGNLLNAEQVEAL--KGKVEFKTKYETRT
FG08935.1 	-GAPPAPDQS---HVEHRVVAKEEKVEYRDQDGNLLDPEEVKAL--EGKVEFKTKYETKT
AN4761.1  	ESQEPSITSS----SIPKIRGTQARVEYRDQQGNILDEAVVDSLRKEGKISIETRHETRT
          	         .       :: . : ::****:.**:*:   *.:* .:**:.::*::**:*

MG02954.1 	RVVDDQGNEILNAVIPPQAEEPKQPVVEAPPAPKNDGPKDPVNPAGVAPPHPDVEG~ADK
NCU01912.1	RVIDANGNEIQ------LGGSGMPKVVRAPKASQP-----------VAPPHPDVQG1VDQ
FG08935.1 	RVVDEQGNEVQ------EPAEGWDQNLGAG----------------VAPPHPDVEG~VDS
AN4761.1  	RLEHGHLVDVVDGK--------------------------------VAPPHPDVEG~QNP
          	*: . :  :: ..                                 ********:*  : 

MG02954.1 	STKQPVKDVAAESLVKDETLSQDGTKEKEQSKAKPASENNEATGASSAAAPAS-------
NCU01912.1	ETPK--IPAAEEVVPQ-AEMSVDGEKEAEEKVAKPASESNEATVSQDIEQSDDVRAAQEK
FG08935.1 	ETVK--GNVEDAAAPQDAAASKDGEKEAEEAKAKPASENQ-----QDATVNDEL------
AN4761.1  	ETQDKPEEIVDDSPASAADAG-SSVGEPNSPEPKPASEGNEATR----------------
          	.* .           .    . ..  * :.  .*****.:.::                 

MG02954.1 	------------------------------------------------------------
NCU01912.1	IQKMVERIQKVANNVDNLKNNVAEKAGDKVDQVEEKAEQAVEKVQEVAEPVEEKAEQVVE
FG08935.1 	------------------------------------------------------------
AN4761.1  	------------------------------------------------------------
          	                                                            

MG02954.1 	------------------------------------------------------------
NCU01912.1	KVQEVAEPAKEKAEQVVEKVQEVAEPAKEKAEQVVEKVQEVAEPAKEKAEEAAEKLAEKV
FG08935.1 	------------------------------------------------------------
AN4761.1  	------------------------------------------------------------
          	                                                            

MG02954.1 	------------------------------------------------------------
NCU01912.1	EEATEQSKEKIESVADNVKEEVTEKAEQVQEKAQEAGEKAQEVVLEKIDQVKEMAEEVGE
FG08935.1 	------------------------------------------------------------
AN4761.1  	------------------------------------------------------------
          	                                                            

MG02954.1 	------------
NCU01912.1	KAEAKTEAKDEL
FG08935.1 	------------
AN4761.1  	------------
          	
```
